# Severe Human Influenza Infections in Thailand: Oseltamivir Treatment and Risk Factors for Fatal Outcome

**DOI:** 10.1371/journal.pone.0006051

**Published:** 2009-06-25

**Authors:** Wanna Hanshaoworakul, James Mark Simmerman, Ubolrat Narueponjirakul, Wiwan Sanasuttipun, Vivek Shinde, Suchada Kaewchana, Darin Areechokechai, Jens Levy, Kumnuan Ungchusak

**Affiliations:** 1 Bureau of Epidemiology, Thailand Ministry of Public Health, Nonthaburi, Thailand; 2 International Emerging Infections Program, Thailand MOPH – U. S. CDC Collaboration, Ministry of Public Health, Nonthaburi, Thailand; 3 Influenza Division, U. S. Centers for Disease Control, Atlanta, Georgia, United States of America; Comprehensive AIDS Reseach Center, China

## Abstract

**Background:**

Influenza is often not recognized as an important cause of severe or fatal disease in tropical and subtropical countries in Southeast Asia. The extent to which Oseltamivir treatment may protect against a fatal outcome in severe influenza infections is not known. Thailand's National Avian Influenza Surveillance (NAIS) system affords a unique opportunity to describe the epidemiology of laboratory-confirmed severe and fatal human influenza infections.

**Methodology/Principal Findings:**

During January 2004 through December 2006, 11,641 notifications to the NAIS were investigated in 73 of 76 Thai provinces. Clinical and demographic data and respiratory swab specimens were collected and tested by PCR for influenza. Using the NAIS database, we identified all patients with laboratory confirmed human influenza (A/H3N2, A/H1N1 and Type B) infection. A retrospective medical record review was conducted on all fatal cases with laboratory confirmed influenza and from a sample of hospitalized cases in 28 provinces. The association of underlying risk factors, Oseltamivir treatment and risk of a fatal outcome were examined. Human influenza infections were identified in 2,075 (18%) cases. Twenty-two (1%) deaths occurred including seven deaths in children less than ten years of age. Thirty-five percent of hospitalized human influenza infections had chest X-ray confirmed pneumonia. Current or former smoking; advanced age, hypertension and underlying cardiovascular, pulmonary or endocrine disease were associated with a fatal outcome from human influenza infection. Treatment with Oseltamivir was statistically associated with survival with a crude OR of .11 (95% CI: 0.04–0.30) and .13 (95% CI: 0.04–0.40) after controlling for age.

**Conclusions:**

Severe and fatal human influenza infections were commonly identified in the NAIS designed to identify avian A/H5N1 cases. Treatment with Oseltamivir is associated with survival in hospitalized human influenza pneumonia patients.

## Introduction

Influenza is a vaccine preventable viral respiratory infection and an important cause of morbidity and mortality in temperate climates, especially among the elderly and the very young [1], [2]. In the US, high quality data on the burden of severe and fatal influenza infection has supported policies to expand vaccination programs [3], [4]. However in tropical and subtropical climates comparatively little information on disease burden is available, especially data describing severe influenza infections that result in hospitalization or death [5]. The non-specific symptoms of influenza infection and the lack of routine laboratory diagnostic testing in most hospitals significantly limits case ascertainment and reporting. In the absence of data on severe and fatal human influenza infections, clinicians and the general public tend to regard such severe infections as rare. The public health impact of this perception can be significant as influenza antiviral medications and vaccines are available but poorly utilized in many countries in East and Southeast Asia [6]. Recently, research from Hong Kong and Singapore using large mortality databases has suggested that influenza-associated mortality rates are similar to those documented in the United States [7]–[9]. In less economically developed countries in East and Southeast Asia, such research is hindered by the lack of robust mortality registries and hospital admission databases. Information on influenza disease burden is essential for policy makers considering investment in control programs.

Outbreaks of avian influenza A/H5N1 virus occurred in poultry in Thailand in late 2003 with human H5N1 cases first identified in early 2004 [10]. In response the Thailand Ministry of Public Health (MOPH) established the National Avian Influenza Surveillance (NAIS) system in 2004. The system's mandate was the identification and prompt investigation of suspected human AI infections in all provinces in all healthcare facilities nationwide [11]. Under NAIS patients presenting with fever and respiratory symptoms were interviewed for the presence of risk factors for H5N1 infection including 1) contact with sick or dead poultry within 7 days before the symptom onset, 2) living in areas where poultry die-offs had occurred within 14 days before symptoms onset and 3) contact with any pneumonia patients in the 10 days before symptoms onset.

If patients had risk factors or the physician suspected avian influenza infection, nasopharyngeal swabs, throat swabs or secretions from endotracheal suctions were collected from patients and tested by RT-PCR for influenza A subtypes H1, H3, H5 and influenza Type B at the Thailand National Institute of Health (NIH) laboratory in Bangkok or at several regional NIH network laboratories [12]. All H5N1 positive specimens and those from rapidly progressive or fatal human influenza cases were confirmed at the central NIH laboratory. Clinical and demographic data were collected as part of case investigations. We analyzed data from the NAIS and medical record reviews to describe the epidemiology of laboratory-confirmed severe and fatal human influenza infections in Thailand. Using a retrospective case-control design, we also examined the association of underlying risk factors, antiviral treatment and risk of a fatal outcome.

## Methods

This research was carried our with approval from and in compliance with the standards of the ethical review committees of the Thailand Ministry of Public Health and the United States Centers for Disease Control and Prevention. During January 2004 through December 2006, the NAIS system recorded 11,641 cases requiring investigation for suspected H5N1 infection reported from 73 of the 76 Thai provinces. All fatal human influenza cases (A(H1N1), A(H3N2) or B) and non-fatal cases hospitalized for two or more days were characterized as severe and eligible for on-site medical record review. Human influenza-associated deaths were patients reported by the NAIS with RT-PCR confirmed influenza infection who died while in the hospital.

**Figure 1:**
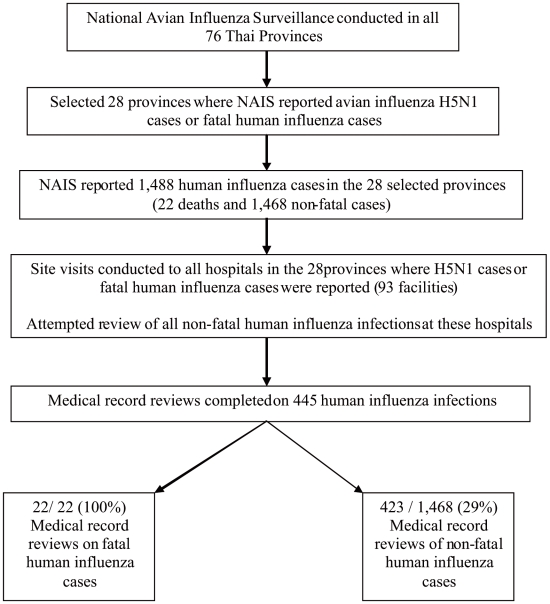
Sample selection process.

We selected the 28 provinces where at least one H5N1 case or human influenza death had been detected by the NAIS. In these 28 provinces, the NAIS recorded 1,488 human influenza infections. We selected all 22 fatal human influenza cases for medical record review. Due to logistical and resource considerations, we sampled a subset of the remaining 1,466 non-fatal human influenza cases using an approach designed to insure that non-fatal cases came from the same population as the fatal cases . (Figure 1) First, we visited all hospitals where a fatal human influenza case had been admitted. In most cases, these patients had been transferred between 2 or 3 hospitals as their clinical course deteriorated. We then attempted to review all nonfatal human influenza cases from these hospitals. In a few cases the hospital clerical staff was not able to locate all of the medical records. Finally, we visited other small rural, district hospitals located in the same provinces which had also reported non-fatal human influenza cases to the NAIS and attempted to review all available records.

Hospitals were contacted in advance and asked to prepare the medical records for review. A standardized 8-page data collection form was used to collect detailed epidemiological and clinical data including underlying illnesses, smoking and HIV status. Two trained study staff jointly conducted each medical record review. Data were entered into a Microsoft Access database. We conducted post data entry validation of all data fields to assure that the electronic record was identical to the paper medical record review form. We examined the crude, unadjusted relationship of individual demographic, epidemiologic and clinical characteristics in relation to risk of death from human influenza. We used multivariable logistic regression to control for age and estimate adjusted odds ratios of a fatal outcome among hospitalized patients with human influenza infection. Specifically, we compared the 22 fatal human influenza cases with the 423 nonfatal human influenza cases with respect to antiviral treatment and underlying conditions (cardiovascular, hypertension, pulmonary, endocrine disorders, renal disease, neurological disorders, chronic gastrointestinal disease, cancer, and hematological disorders).

Insufficient numbers of subjects were observed to have gastrointestinal disease, cancer, hematological disorders, neurological disorders, renal disease, aspirin use or HIV-positive status to evaluate the association of these conditions with a fatal outcome. Furthermore, small numbers precluded evaluation of more than two risk factors at a time. We also examined the association of fatal outcome with age, gender, smoking status, and antiviral treatment among human influenza cases. We calculated odd ratios and 95% confidence intervals (CI) using SAS 9.1 software. We used exact logistical regression methods to calculate adjusted odds ratios (ORs). Variables were coded using an indicator variable for each category level except for the referent level. The 10–19 year age group was selected as the reference category after explanatory analysis revealed this category to have the lowest risk of death. Similarly, current and former smoking was coded with indicator variables with no smoking designated as the referent category.

## Results

**Figure 2:**
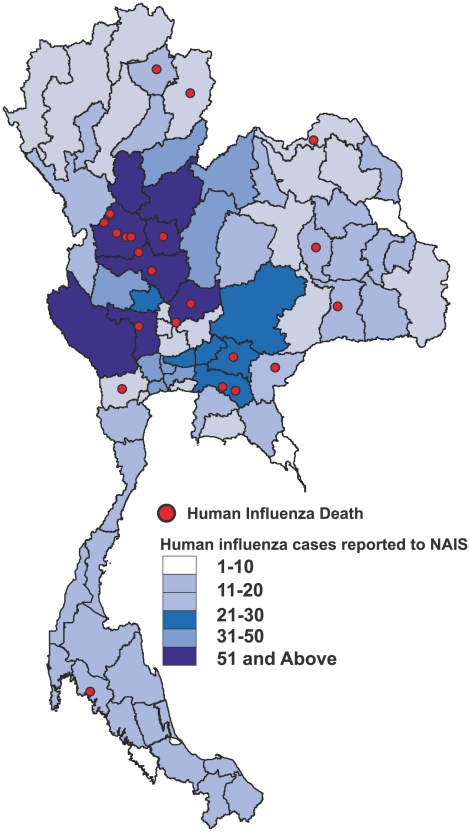
Geographic distribution of human influenza cases.

In 2004 the NAIS recorded 2,846 case investigations including 106 deaths nationwide. In 2005 there were 3,199 investigations and 158 deaths while in 2006 there were 5,596 investigations with 177 deaths. In this population of 11,641 persons, 2,075 (18%) laboratory-confirmed human influenza infections and 22 influenza-associated deaths were identified. Human influenza infections were identified in 73 of the 76 Thai provinces while H5N1 infections were localized to the central and northern regions where most commercial poultry production is located. (Figure 2) In total, we reviewed medical records from 445 human influenza cases admitted to 93 hospitals in 28 Thai provinces. Information on comorbidities was missing on only three individuals, one of which was a fatality. The mean age of sampled cases was 22 years (median 13) and 57% were male. Very young children and the elderly were underrepresented in the study sample compared to other populations under active pneumonia surveillance in Thailand [13], [14]. In 2004, subtype A/H1N1 accounted for 36%, A/H3N2 for 38% and Influenza type B accounted for 27% of all infections. In 2005, 50% of human influenza infections were due to A/H3N2 and 43% were due to Influenza B, while the A/H1N1 subtype caused 79% of infections in 2006. (Table 1)

**Table 1:** Human influenza cases and deaths reported by National Avian Influenza Surveillance.

	2004			2005			2006		
	Reported Cases (%)	Reviewed	Deaths^1^	Reported Cases (%)	Reviewed	Deaths	Reported Cases (%)	Reviewed	Deaths
A/H1	104 (36)	27 (40%)	1	40 (8)	14 (10%)	1	981 (79)	178 (74%)	5
A/H3	110 (38)	28 (42%)	1	264 (50)	87 (63%)	5	108 (9)	28 (12%)	2
Type B	78 (27)	12 (17%)	1	230 (43)	37 (27%)	3	160 (13)	34 (14%)	3
Total	292	67	3	534	138	9	1249	240	10

1 All deaths also reviewed.

Influenza A/H3N2 caused 8 deaths, subtype A/H1N1 caused 7 deaths and type B caused 7 deaths. Seven (32%) of deaths occurred in children less than 10 years of age, 5 (23%) in patients aged 10–49 years and 10 (45%) of deaths occurred in patients 50 years of age or older. Fifteen (71%) fatal cases had at least one underlying illness. The most frequently documented underlying illness was hypertension (6), followed by cardiovascular disease (4), pulmonary disease (4) and endocrine disorders (4). Twenty-one (95%) of the fatal cases were diagnosed with a lower respiratory tract infection (LRTI). The median time from onset of illness until death was 6.5 days (mean 10.7, range 1–68 days). Five (22%) patients with fatal infections had bacterial co-infections reported including 2 cases with *Burkholderia pseudomallei*, 2 cases with *Pseudomonas aeruginosa* and 1 *Staphylococcus aureus* coinfection. Among the 423 nonfatal human influenza infections, 340 (80%) were diagnosed with an LRTI. The median duration of illness was 5 days (1–22 days) and the median length of hospital stay was 3 days (range 1–21). There were 150 (35%) chest X-ray confirmed pneumonia cases and 3 of these required endotracheal intubation and intensive care. Among those with a diagnosis of LRTI, 41 (27%) were children 0–5 years while 10 (7%) cases were ≥60 years of age.

The age distribution of sampled cases was slightly older than the non-sampled cases but the gender distribution was similar. (Table 2) Compared to nonfatal cases, cases with a fatal outcome were more likely to have an underlying cardiovascular disease, hypertension, chronic pulmonary disease, and endocrine disorders although only hypertension remained significant after adjustment for age. (Table 3) Death was associated with female gender but this did not reach statistical significance. Previous or current smoking was significantly associated with a fatal outcome but this did not reach statistical significance after controlling for age. Death was associated with advanced age and the 10–19 year age group experienced the fewest fatal cases.

**Table 2:** Age and gender distribution of sampled cases compared to all cases reported to NAIS in 28 provinces.

Variables	Sampled Cases (#, %)	All Other NAIS Cases (#, %)	Total in 28 Provinces
**Age**			
0–4	81 (18)	182 (17)	263
5–9	98 (22)	285 (27)	383
10–19	101 (23)	238 (23)	339
20–59	135 (30)	302 (29)	437
60+	30 (7)	36 (3)	66
	Chi Square for Age Distribution P = 0.02		
**Gender**			
Male	259 (58)	574 (55)	833 (56)
Female	186 (42)	469 (45)	655 (44)
	Chi square for Gender Distribution P = 0.26		
Total	445	1043	1488

**Table 3:** Factors associated with a fatal outcome.

	Outcome			Unadjusted		Age Adjusted	
	Fatal	Non-Fatal		OR	95% CI	OR*	95% CI
Year							
2004	3 (14%)	64(15%)		1.08	0.29–4.03	1.21	0.20–5.09
2005	9 (41%)	129 (31%)		1.60	0.64–3.80	1.66	0.57–4.81
2006	10 (45%)	230(54%)		1	ref	1	ref
		chi-sq	0.59				
Antiviral Treatment							
No Oseltamivir	17 (77%)	113 (27%)		1	ref	1	ref
Oseltamivir	5 (23%)	310 (73%)		0.11	0.04–0.30	0.13	0.04–0.40
		chi-sq	<0.0001				
Comorbidities							
No Cardiovascular disease	17 (81%)	408 (97%)		1	ref	1	ref
Cardiovascular disease	4 (19%)	13 (3%)		7.38	2.18–25.04	4.04	1.08–15.11
		chi-sq	0.0002				
No Hypertension	15 (71%)	407(97%)		1	ref	1	ref
Hypertension	6 (29%)	14 (3%)		11.63	3.92–34.46	4.67	1.03–20.85
		chi-sq	<0.0001				
No Chronic pulmonary disease	17 (81%)	401 (95%)		1	ref	1	ref
Chronic pulmonary disease	4 (19%)	20 (5%)		4.72	1.45–15.32	3.11	0.64–11.76
		chi-sq	0.0048				
No Endocrine disorder	17 (81%)	414 (98%)		1	ref	1	ref
Endocrine disorder	4 (19%)	7 (2%)		13.92	3.71–52.13	5.01	0.84–26.56
		chi-sq	<0.0001				
Gender							
Male	10 (45%)	249 (59%)		1	ref	1	ref
Female	12 (55%)	174 (41%)		1.72	0.73–4.06	1.7	0.63–4.55
		chi-sq	0.21				
Tobacco							
Never	15(75%)	385(93%)		1	ref	1	ref
Current	3(15%)	18(4%)		4.3	1.1–16.1	1.93	0.28–10.01
Former	2(10%)	10(2%)		5.1	1.0–25.5	2.08	0.17–14.28
		chi-sq	0.006				
Age							
0 to 9	7 (32%)	172 (41%)		4.1	0.5–33.6	-	
10 to 19	1 (5%)	100 (24%)		1	ref		
20–49	4 (18%)	99 (23%)		4.0	0.4–36.8	-	
50 plus	10 (45%)	52 (12%)		19.2	2.4–154.4	-	
		chi-sq	0.0001				

* Age Adjusted ORs calculated using Exact Logistic Regression methods.

Sixty-three percent of patients were treated with Oseltamivir in 2004, 64% in 2005 and 77% in 2006 (p = .01). Overall, 5 (1.5%) of 318 patients treated with Oseltamivir died compared with 17 (5%) fatal outcomes among 131 patients who did not receive treatment. Antiviral treatment with Oseltamivir was statistically associated with survival with a crude OR with Oseltamivir treatment of .11 (95%CI: 0.04–0.30) and .13 (95%CI: 0.04–0.40) after controlling for age. Oseltamivir was also associated with survival when controlling for the potential confounding variables cardiovascular disease (OR 0.13; 95% CI 0.04–0.38) and hypertension (OR 0.14; 95% CI 0.04–0.44) (data not shown). The mean time from onset of symptoms to initiation of Oseltamivir treatment in the five fatal cases was 4 days (median 4, range 2–7,) compared to 2.62 days (median 2, range 0–13) in the 303 surviving patients. Two of the 5 fatal cases who received Oseltamivir treatment died within 2 days of hospital admission.

## Discussion

In response to concerns regarding A/H5N1 and the raised pandemic threat, Thailand's NAIS system was rapidly established with a high-quality laboratory diagnostic system that enabled the country to quickly identify a small number of human AI cases among thousands of suspected infections [12]. While each new case of AI received global media attention, the NAIS system identified approximately 80 times as many human influenza infections. More deaths resulted from these infections than from H5N1 (22 versus 17). The surveillance population under the NAIS system was restricted mainly to persons with acute respiratory illness who had a recent history of exposure to sick or dying poultry. In this limited population of 11,641 persons, 2,075 cases of human influenza were identified. Twenty-two deaths were identified that would have otherwise gone unrecognized by the existing passive surveillance system for human influenza. In contrast to the NAIS, between 1999 and 2003 the Thailand national routine passive surveillance system reported only 3 deaths due to human influenza infection. During 2004–2006, a single influenza-associated death was reported through routine passive surveillance in the Thai population of 65 million persons [15]. This under-ascertainment can be explained by the lack of laboratory diagnostic capacity for influenza in nearly all public hospitals and to a common misperception among Thai clinicians that influenza is neither common nor severe.

The NAIS case definition included exposure to sick or dying poultry and consequently very young children and the elderly were underrepresented in our sample. As these age groups are at increased risk for serious complications, the NAIS would not have captured many severe and fatal human influenza infections. Our sampling strategy involved sampling non-fatal cases from the same provinces where fatal human influenza cases (or H5N1 cases) had been reported to best approximate the population from which fatal cases arise. This approach resulted in some small though statistically significant differences in the age structure between sampled and non-sampled cases. Potential variability in the age distribution of the population that each hospital serves could explain these differences.

We observed a statistically significant survival benefit associated with Oseltamivir treatment among patients hospitalized with influenza. This potentially important finding should be interpreted with caution. Although Oseltamivir has been shown to reduce symptom duration, complications and hospitalization among adult outpatients as well as decrease lower respiratory tract infections in observational studies [16]–[20], evidence of a survival benefit from Oseltamivir treatment for human influenza infection has been infrequently reported [21]. Our small, retrospective, observational study has limitations with respect to establishing causality and we lacked additional information such as functional status and severity of illness scores which may have confounded or biased our results. However, the strength and significance of these data indicate a survival benefit from Oseltamivir treatment in hospitalized influenza patients. More research is needed to verify these findings which could influence pneumonia case management approaches as well as strategies for antiviral use during a pandemic.

Outside of Singapore and Hong Kong, no epidemiological studies of human influenza-associated mortality have been published in Southeast Asia [5]. The 22 deaths we uncovered probably represent only a small fraction of human influenza-associated mortality in Thailand during 2004–2007 given the narrowly defined criteria for testing based on risk factors for H5N1. Although data on influenza–associated mortality in Thailand are not available, a few recent studies provide insight into the contribution of influenza to inpatient pneumonia and outpatient febrile respiratory illness in Thailand [22], [23]. While the precise figure varies from year to year, approximately 8–10% of hospitalized pneumonia cases in Thailand are attributed to human influenza infection [24], [25]. In our study, 35% of all hospitalized influenza cases had been diagnosed with chest X-ray confirmed pneumonia. Studies from other regional countries report that 11–26% of outpatients with febrile respiratory illness have influenza virus infections [5].

In conclusion, our findings suggest that the national passive surveillance system substantially underreports the burden of severe and fatal human influenza infection in Thailand and that treatment with Oseltamivir may provide a survival benefit in these patients. The elderly, smokers and persons with underlying comorbidities with human influenza infection are at increased risk of a fatal outcome. Further research on influenza-associated mortality and Oseltamivir treatment using national hospital discharge, mortality and influenza surveillance databases is needed to inform influenza control policies, guide clinical management and support pandemic preparedness planning.
